# Influence of Green Leafy Vegetables in Diets with an Elevated ω-6:ω-3 Fatty Acid Ratio on Rat Blood Pressure, Plasma Lipids, Antioxidant Status and Markers of Inflammation

**DOI:** 10.3390/nu11020301

**Published:** 2019-01-31

**Authors:** Melissa Johnson, Wendell H. McElhenney, Marceline Egnin

**Affiliations:** 1 College of Agriculture, Environment and Nutrition Sciences, Tuskegee University, Tuskegee, AL 36088, USA; 2 Department of Agricultural and Environmental Sciences, College of Agriculture, Environment and Nutrition Sciences, Tuskegee University, Tuskegee, AL 36088, USA; wmcelhenney@tuskegee.edu (W.H.M.); megnin@tuskegee.edu (M.E.)

**Keywords:** collard greens, purslane, sweetpotato greens, ω-6:ω-3 fatty acid ratio, cardiovascular disease, spontaneously hypertensive rat

## Abstract

The typical Western dietary pattern has an elevated ω-6:ω-3 fatty acid ratio (FAR), which may exacerbate the risk of chronic disease. Conversely, the consumption of diets containing green leafy vegetables (GLVs) have been demonstrated to attenuate disease risk. This study investigated the effects of collard greens (CG), purslane (PL) and orange flesh sweetpotato greens (SPG) on measures of disease risk in rats fed diets with a 25:1 ω-6:ω-3 FAR. Male spontaneously hypertensive rats (SHRs) were randomly assigned to four dietary groups (*n* = 10/group) with a 25:1 ω-6:ω-3 FAR. Experimental diets contained 4% (dried weight) CG, PL or SPG. Dietary intake, body weight, blood pressure, plasma adiponectin, high sensitivity C-reactive protein (hsCRP), oxygen radical absorbance capacity and lipid profile were determined using standardized procedures. Following a 6-week consumption period, systolic blood pressure, plasma adiponectin, total and low-density lipoprotein (LDL) cholesterol decreased following the consumption of diets containing GLVs. While hsCRP increased in SHRs fed diets containing CG and PL, plasma antioxidant capacity was significantly reduced (*p* < 0.05) with the consumption of diets containing the GLVs. These findings suggest that CG, PL and SPG have the potential to decrease risks for cardiovascular disease (CVD) associated with the consumption of diets with an elevated ω-6:ω-3 FAR.

## 1. Introduction

Hypertension, one of the most common forms of cardiovascular disease (CVD), significantly contributes to morbidity and mortality in the United States as well as globally [1]. Consumption of the Western dietary pattern, characterized by increased intakes of processed foods, animal products and relatively minimal intakes of whole grains, fruits and vegetables, is related to increased risk of hypertension and other CVDs and associated comorbidities [2,3,4,5,6,7]. Diets rich in omega-6 (ω-6) fatty acids together with the deficiency of omega-3 (ω-3) fatty acids, leading to an elevated fatty acid ratio (FAR), further increases the atherogenicity related to increased disease risk [8,9]. The current ω-6/ω-3 FAR within the Western diet is estimated to be approximately 25:1 [10,11]. This imbalance in the ω-6/ω-3 FAR, coupled with traditional Western dietary practices, further exacerbates the increased risk for hypertension and other CVDs [12].

Risks associated with hypertension and other CVDs may be minimized by engaging in more prudent dietary practices and increased consumption of diets that contain fruits, vegetables, whole grains and fatty fish [13,14,15,16]. Although Americans consume less than recommended intakes [17,18], inclusion of cruciferous and dark green, leafy vegetables (GLVs) into the diet is particularly emphasized, as consumption has been demonstrated to mitigate the risks associated with disease pathogenesis and mortality [19,20,21,22]. Collard greens (*Brassica oleracea*), a traditional GLV consumed in the southeastern region of the United States, as well as purslane (*Portulaca oleracea*) and sweetpotato greens (*Ipomoea batatas* L.), nontraditional GLVs are established sources of antioxidant and bioactive compounds that exhibit potent free radical scavenging and antioxidant capabilities [23,24]. The nutritional profile of collard greens, purslane and orange flesh sweetpotato greens suggest their potential functionality in disease prevention and health promotion [25,26,27,28,29,30,31].

In light of the potential benefits of collard greens, purslane and sweetpotato greens, coupled with gaps in knowledge regarding their contribution to health promotion and disease prevention in vivo, research studies are warranted to affirm their influence on disease risk. Therefore, the purpose of this research study was to determine the influence of collard greens, purslane and orange flesh sweetpotato greens, in diets with an ω-6/ω-3 FAR, reflective of the typical Western dietary pattern (i.e., 25:1), on body weight, systolic blood pressure, plasma adiponectin, high sensitivity c-reactive protein, oxygen radical absorbance capacity and lipid profile of the spontaneously hypertensive rat. Furthermore, by expanding our knowledge of these GLVs and their prospective role as functional foods in disease prevention, dietary recommendations for additional cardiometabloic health and protection may emerge. 

## 2. Materials and Methods 

### 2.1. Dietary Formulations 

The American Institute of Nutrition (AIN)-76A purified rodent diet was modified to a final ω-6/ω-3 FAR of 25:1; experimental diets included 4% collard greens, purslane and orange flesh sweetpotato greens powder, respectively (Table 1). Collard greens (purchased from the local farmer’s market, Tuskegee, AL, USA), purslane and sweet potato greens (purchased from the International Farmer’s Market, Duluth, GA, USA) were freeze-dried for approximately 48 hours (Virtis Genesis 25SL, Gardiner, NY, USA) and powdered prior to the manufacturing process. The unmodified AIN-76A diet served as the standardized control. Control and experimental diets were formulated to be isonitrogenous and isocaloric. in conjunction with recommendations set forth by the National Cholesterol Education Program Expert Panel for carbohydrate (50–60% of total calories), protein (~15% of total calories) and fat (25–35% of total calories- less than 7% saturated fat; up to 10% polyunsaturated fat; up to 20% monounsaturated fat) (Table 2). The Division of Land O’Lakes Purina Feed, LLC (Purina TestDiet®, Richmond, IN, USA), manufactured diets; NP Analytical Laboratories (St. Louis, MO, USA) confirmed specifications. 

### 2.2. Animal Feeding 

Four-week-old male, spontaneously hypertensive rats (SHRs, *n* = 50), weighing approximately 60 grams were housed individually in polypropylene cages and maintained on a 12:12 hour light-dark photoperiod cycle, in a controlled environment (20–22 °C; 50–55% relative humidity) with ad libitum access to water, rodent chow (three days) and AIN-76A purified rodent diet (seven days) during the 10 days acclimation period. SHRs were randomly assigned to one of five dietary groups (AIN-76A (standardized control), control, CG, PL or SPG) and consumed the diets for 6 weeks. Animals were pair-fed according to the average dietary intake of SHRs assigned to diets containing GLVs. Food intake and body weight were measured daily and once a week, respectively. Following the completion of the six weeks feeding trial, SHRs were anesthetized using a Ketamine/Acepromazine combination cocktail (75–100 mg/kg body weight) and subsequently euthanized via the over-inhalation of carbon dioxide. Blood was collected via cardiac puncture; SHR organs were removed and stored at −80 °C prior to analysis. The Tuskegee University Animal Care and Use Committee (Tuskegee, AL 36088) approved the protocols involved in the care and use of animals for this research study, in accordance with standards established by the National Institutes of Health. 

### 2.3. Systolic Blood Pressure

Weekly systolic blood pressure measurements were measured utilizing the noninvasive tail cuff blood pressure (NIBP) system (ML 125/M, ADInstruments, Inc., Colorado Springs, CO, USA) according to manufacturer’s instructions. 

### 2.4. Adiponectin and hsCRP

Plasma adiponectin and high sensitivity C-reactive protein (hsCRP) concentrations were determined using the adiponectin rat ELISA kit (Abcam®, Cambridge, MA, USA) and the rat C-reactive protein ELISA kit (Helica Biosystems, Santa Ana, CA, USA), respectively. Adiponectin and hsCRP concentrations were estimated based on optical density values obtained from standard curves, measured at 450 nm using a BioTek® Microplate Reader (Winooski, VT, USA). 

### 2.5. Antioxidant Capacity

SHR plasma antioxidant capacity was determined using the OxiSelect Oxygen Radical Antioxidant Capacity (ORAC) activity kit (Cell Biolabs, San Diego, CA, USA) according to manufacturer’s protocol. ORAC values, expressed as μMoles of TroloxTM Equivalents (TE) were calculated based on the TroloxTM antioxidant standard curve. The area under the curve (AUC) was calculated using the following equation

AUC= 1 + RFU5/RFU0 + RFU10/RFU0 + …… RFU55/RFU0 + RFU60/RFU0(1)

where, RFU0 = relative fluorescence value at time point zero and RFUx = relative fluorescence value at time points (e.g., minute 5, minute 10 …… minute 55, minute 60).

### 2.6. Lipid Profile: Plasma Triglycerides, Total Cholesterol, High-Density Lipoprotein- Cholesterol (HDL-C) and Low-Density Lipoprotein-Cholesterol (LDL-C) + Very Low-Density Lipoprotein-Cholesterol (VLDL-C)

Plasma total cholesterol (Abcam® Inc., Cambridge, MA, USA), triglyceride (Abcam® Inc., Cambridge, MA, USA), HDL-C (Abcam® Inc., Cambridge, MA, USA) and LDL-C + VLDL-C (Abcam® Inc., Cambridge, MA, USA) concentrations were determined using assay kits, according to manufacturer’s instructions. Sample and standard(s) optical density values were measured at 450 nm using a BioTek® Microplate Reader (Winooski, VT, USA). 

### 2.7. Statistical Analysis

Data are presented as mean + SEM. Statistical analyses were performed using the GLM procedure (SAS Institute, Inc., Cary, NC, USA). When the omnibus F test was declared significant, Duncan’s procedure was used to compare group means. The level of significance was *p* < 0.05.

## 3. Results

### 3.1. Dietary Intake, Body Weight

No differences (*p* > 0.05) in dietary intake throughout the duration of the feeding study were observed among SHRs (Table 3). Although there were similar average dietary intakes at week 1 (initial dietary intake) and total dietary intakes among SHRs, SHRs assigned to the PL dietary group consumed slightly more than those assigned to the other dietary groups at week 6. While SHRs assigned to the different dietary groups had similar body weights at baseline and during the commencement of the feeding study, at week 6 groups fed diets with a 25:1 ω-6/ω-3 FAR were heavier (*p* < 0.05) than those consuming the AIN-76A diet. 

### 3.2. Systolic Blood Pressure

**Figure 1:**
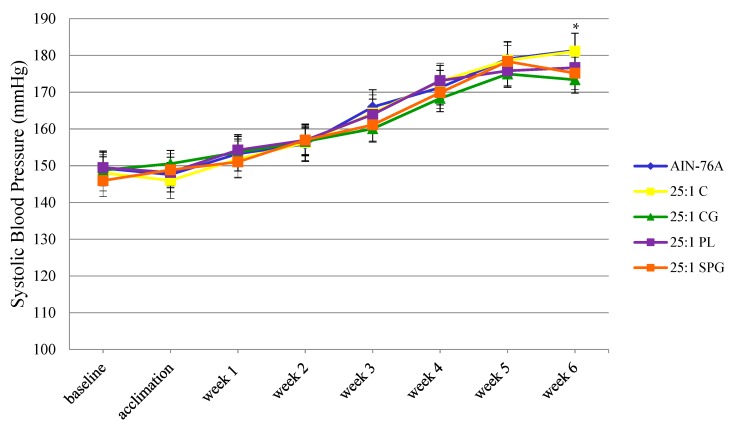
Mean systolic blood pressure of SHRs consuming the AIN-76A diet and diets with a 25:1 ω-6/ω-3 FAR for 6 weeks. * collard greens diet was statistically different (*p* < 0.05) than the AIN-76A and control diets.

Beginning at week 3, average systolic blood pressure decreased in SHRs consuming diets containing CG, PL and SPG in comparison to those consuming the AIN-76A and control diets (Figure 1). At week 6, consumption of the CG (173.4 mmHg) diet resulted in a decrease (*p* < 0.05) in systolic blood pressure compared to the AIN-76A (181.4 mmHg) and control (181.1 mmHg) diets. Among SHRs consuming diets containing GLVs, CG were able to modulate slightly greater non-significant decreases in systolic blood pressure in comparison to PL and SPG. 

### 3.3. Adiponectin and hsCRP

SHR plasma adiponectin and hsCRP concentrations are presented in Table 4. Plasma adiponectin levels were significantly reduced (*p* < 0.05) among SHRs consuming the PL (29.5 μg/mL) diet versus those consuming the AIN-76A (43.0 μg/mL) and C (38.6 μg/mL) diets. Although not statistically significant, plasma adiponectin levels were reduced among SHRs assigned to the CG (35.1 μg/mL) and SPG (31.5 μg/mL) dietary groups. Among SHRs assigned to the different dietary groups, the lowest hsCRP levels were present in the plasma of those consuming the SPG diet (1084.2 μg/mL) followed by those consuming the C diet (1092.2 μg/mL). Diets containing CG (1164.0 μg/mL) and PL (1452.0 μg/mL) resulted in decreased plasma hsCRP concentrations.

### 3.4. Antioxidant Capacity

**Figure 2:**
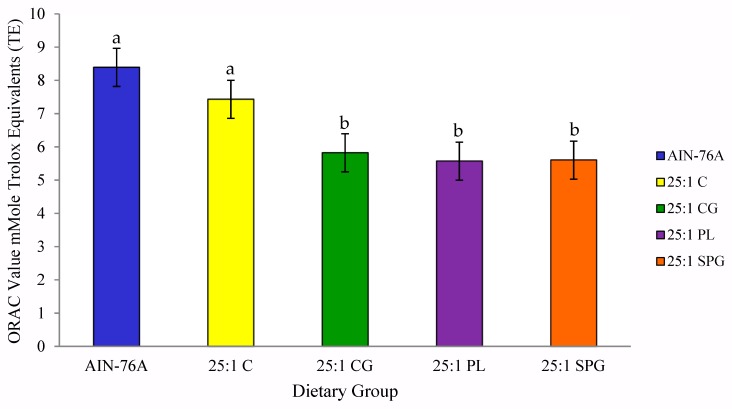
Mean plasma ORAC concentration of SHRs consuming the AIN-76A diet and diets with a 25:1 ω-6/ω-3 FAR for 6 weeks. Results are presented as mean ± SEM mMole TE/L of 10 SHRs per dietary group; bars with different alphabetical superscripts indicate statistical significance at *p* < 0.05 according to Duncan’s post hoc test values.

The antioxidant capacity of SHR plasma was significantly reduced following the consumption of the CG (5.8 mMole/TE), PL (5.6 mMole/TE) and SPG (5.6 mMole/TE) diets (Figure 2).

### 3.5. Lipid Profile

In comparison to the control diet (97.0 mg/dL), triglyceride levels were increased among SHRs consuming the PL (113.0 mg/dL) and SPG (118.4 mg/dL) diets and decreased following the consumption of the CG diet (92.2 mg/dL) (Table 5). Although not significant, total cholesterol and LDL-C + VLDL-C levels were decreased among SHRs consuming the CG, PL and SPG diets in comparison to the AIN-76A and control diets. In comparison to the control diet (33.7 mg/dL), levels of HDL-C were increased among SHRs consuming the CG (38.7 mg/dL) and PL (41.3 mg/dL) diets. 

## 4. Discussion

The imbalance in the ω-6/ω-3 FAR (e.g., 25:1), as seen in traditional Western dietary practices, further exacerbated the increased risk of hypertension and other CVDs risk factors as demonstrated in the present study. Throughout the duration of the study, as well as at the conclusion of the research, SHRs consuming the control, CG, PL and SPG diets weighed significantly more than those consuming the AIN-76A diet, possibly explained by the caloric density of the AIN-76A diet. AIN-76A diet had fewer calories per 100 grams and less than 3 times the amount of total fat. The ability of diets containing CG, PL and SPG to promote an attenuation in systolic blood pressure corroborate previous research demonstrating the ability of cruciferous and green, leafy vegetables to reduce blood pressure and reduce the risks associated with CVD [32,33,34]. The reduction in blood pressure is probably attributed to the vasodilative and subsequent antihypertensive effects of the antioxidant compounds such as quercetin, which is commonly found in these vegetables [35,36,37,38].

Levels of adiponectin, an adipose-specific protein, are inversely associated with levels of adiposity [39]. Consequently, lower levels of adiponectin are linked to increased risk for obesity, insulin resistance, diabetes, cardiovascular and other diseases [40,41,42]. Increases in plasma adiponectin concentrations have been observed with the obstruction of the renin-angiotensin system [43], increased HDL concentrations, and decreased body mass index [44]. The consumption of purslane seeds for 16 weeks resulted in significant decreases in blood glucose, LDL cholesterol, total cholesterol and triglycerides and a significant increase in HDL cholesterol [45]. Additionally, purslane has been demonstrated to reduce the risks associated with oxidative stress, cardiovascular disease and other diseases [46,47]. In a study by Hussein purslane extract incorporated into a high-fat diet was able to inhibit weight gain and improve insulin resistance [48]. Although adiponectin concentrations were not measured in this study, one would anticipate increased levels of adiponectin based on its relationship with the parameters studied. 

In the present study, plasma adiponectin was not increased in SHRs consuming diets containing CG, PL and SPG. Research suggests a positive relationship with omega-3 fatty acid supplementation and adiponectin, with adiponectin levels increasing with increasing omega-3 fatty acid intake [49,50]. Furthermore, it has been suggested that the risk for obesity increases with an increase in the omega-6/omega-3 fatty acid ratio [51]. The elevated omega-6/omega-3 fatty acid ratio in the current study may in part explain the reductions in plasma adiponectin. Dietary fat is hypothesized to decrease adiponectin levels by increasing susceptibility to weight gain, obesity and inflammation [52,53,54]. While decreases in plasma adiponectin concentrations have been reported with increased dietary fat [55], others have reported plasma adiponectin concentrations to be positively associated with total dietary fat intake [56]; high fat intakes have also been reported to exert no influence on adiponectin concentrations [57,58]. Consequently, research findings concerning the relationship between dietary fat and adiponectin concentrations are inconclusive. Although some of current findings of this study were not in agreement with previous research, it is hypothesized that longer term feeding of diets may enhance the clinical cardioprotecive effects of the GLVs within diets with an elevated omega-6/omega-3 fatty acid ratio.

In addition to dietary fat, the dietary fiber and antioxidant compounds contained in CG, PL and SPG may have influenced SHR plasma adiponectin concentrations. Research has affirmed that increased diet quality (e.g., increased consumption of whole grains, fruit, vegetables, nuts/legumes, long-chain fats, and PUFAs) favorable influences plasma biomarkers such as adiponectin [59]. Increased consumption of fruits and vegetables, rich plant sources of dietary antioxidants, have been connected with increased antioxidant concentrations associated with increased adiponectin concentrations [60] as well as decreased central adiposity and oxidative stress. The importance of individual dietary constituents acting in synergy during nutrient metabolism, more specifically dietary fiber and dietary antioxidants, has recently been highlighted as dietary fiber may act as a carrier of antioxidants and assist in transport [61]. However, in the current study the mechanisms and effectiveness of dietary fiber as a carrier for dietary antioxidants and subsequent influence on adiponectin concentrations were not determined. Furthermore, dietary fiber has been demonstrated to significantly interact with the adiponectin gene polymorphism to influence adiponectin concentration, with GG homozygotic individuals displaying significantly greater adiponectin concentrations, even with low fiber intake [62]. In addition, it has been indicated that the total antioxidant capacity of the diet is related to central adiposity and disease risk, with individuals having greater dietary antioxidant capacities exhibiting less central adiposity and disease risk (i.e., higher HDL-C, lower triglyceride concentration, total cholesterol: HDL-C ratio and LDL-C) [63]. Because diets containing GLVs contained greater dietary fiber and antioxidant concentrations, it would be expected that these diets would elicit significantly greater antioxidant and adiponectin concentrations in the SHR as well. Unfortunately, our findings did not meet this expectation.

In addition, increased plasma hsCRP levels among SHRs consuming the PL and SPG diets may be related to lower plasma adiponectin concentrations. The ability of adiponectin to regulate CRP synthesis has been demonstrated, with higher levels of adiponectin suppressing the synthesis of CRP in endothelial cells [64]. Research indicates higher dietary antioxidant capacities to be positively associated with increased adiponectin levels [65] and decreased CRP levels [66]. Higher levels of (dietary) antioxidants are believed to indirectly increase adiponectin concentrations by decreasing oxidative stress, which reduces the expression of adiponectin [67]. The lower plasma antioxidant capacities, as shown in this research, among SHRs consuming diets containing GLVs- rich sources of antioxidant compounds, versus those consuming diets void of GLVs may be attributed to factors such as decreased antioxidant bioavailability (e.g., interactions with other nutrients and components of the food matrix) and the physiological status of the SHRs [68,69]. 

Kahlon et al., demonstrated the ability of CG to exert a hypocholesterolemic effect in vitro, the influence of which was significantly enhanced following steam cooking [70,71]. The observed hypocholesterolemic ability of CG in this research is believed to be attributed to antioxidant compounds (e.g., sulforaphane, isothiocyanates) and other nutrient fractions, as well as physical and chemical conformational changes that influence hydrophobicity, active binding sites and the stimulation of the synthesis of detoxifying enzymes that facilitate the binding and excretion of bile acids. The increased fecal excretion of bile acids, reductions in both serum and liver total cholesterol and reduced liver triglycerides have been observed in male Sprague-Dawley rats fed cholesterol-free diets containing 5% SPG for 4 weeks; all observations were statistically significant with the exception of serum total cholesterol [72]. Besides the polyphenol and sterols present in SPG, insoluble dietary fibers and water-soluble viscous polysaccharides are suggested to participate in the hypocholesterolemic process. 

In the present study, feeding CG, PL and SPG all exerted a non-significant hypocholesterolemic effect; a non-significant hypotriglycemic effect was observed following the consumption of the CG diet. The hypocholesterolemic effects of vegetables may be explained in part by the presence of dietary fibers, which bind bile acids for excretion, stimulate the conversion of free cholesterol to bile acid(s) and impede cholesterol synthesis [73,74]. The increased HDL-C levels following the consumption of diets containing CG and PL, although not statistically significant, are in agreement with findings correlating vegetable consumption to increased HDL-C levels [75]. Furthermore, increased consumption of dietary fiber from vegetable products has been associated with decreased total cholesterol, plasma C-reactive protein and LDL-C, in addition to increased HDL-C and decreased risk for cardiovascular and other diseases [76,77]. This is of particular significance as HDL-C is inversely related to CVD risk and mortality, with individuals with higher HDL-C levels often demonstrating lower disease risk and mortality [78]. Other research has revealed the ability of CG, PL and SPG to influence the erythrocyte fatty acid profile of spontaneously hypertensive rats [79].

Reductions in systolic blood pressure and total cholesterol among SHRs consuming diets containing GLVs in this study suggest the potential mediation of these parameters by tissue omega-3 fatty acids. Although findings have been inconclusive, generally increasing dietary omega-3 fatty acids have been associated with decreased risk for CVD risk, exhibited in influences on hsCRP, triglycerides, LDL-C and HDL-C [80]. Mechanisms by which omega-3 fatty acids reduce CVD risk include mediating eicosanoid metabolism and gene expression, increased endothelial relaxation, as well as decreasing platelet aggregation, triglyceride levels, blood pressure [81]. However, many of these mechanisms remain unclear. 

Although the current research study did not focus on the mechanisms whereby which the nutritional, chemical, antioxidant and bioactive compounds within the GLVs were able to mitigate the risks for CVD, several metabolic pathways may be initiated or suppressed that may to some extent offer insight to the observed findings. For example, reductions in oxidative stress, inflammation, blood pressure and improved endothelial function may be mediated by compounds such as nitrates [82], phytochemicals [83], flavonoids [84], polyphenols [85] and omega-3 fatty acids [86], which are commonly found in GLVs such as collard greens, purslane and sweet potato greens. While brown and white adipose tissue levels were not measured, research has demonstrated that these tissues play a vital role in lipid storage, endocrine function, adipokine concentrations and inflammation [87]. Understanding the functioning and mechanisms of specific compounds within these GLVs may also potentially provide awareness regarding the differential effects of GLVs on lipid profile (e.g., TC, TAG, HDL-C, LDL-C), oxidative status and adipokine concentrations. 

## 5. Conclusions

The increased risk for high blood pressure and other cardiovascular diseases, the leading cause of morbidity and mortality in the United States, is associated with the consumption of diets rich in ω-6 fatty acids and other atherogenic dietary components (e.g., excessive saturated fats, trans fats, cholesterol, and sodium). This research study examined the influence of traditional and nontraditional GLVs on disease risk, when incorporated into diets with an ω-6: ω-3 FAR reflective of the typical American diet (i.e., 25:1). The ability of these GLVs to favorably modulate blood pressure and lipid metabolism within an animal model predisposed to developing hypertension was made evident. Dietary fibers, antioxidant compounds and ω-3 fatty acids contained in these GLVs are believed to act in synergy to modulate blood pressure, gene expression, inflammatory process, lipid and lipoprotein concentrations. These facts lead to the question of the impact of other ω-6: ω-3 FARs on the same metabolic parameters measured in the study. 

As the SHR is an animal model commonly employed to investigate the mechanisms of high blood pressure pathogenesis and progression and extrapolation to humans, the findings of this research may have implications for human health. Based on past and current research findings, particular emphasis should be placed on the inclusion of collard greens, purslane and sweet potato greens into an integrative dietary intervention to prevent high blood pressure, dyslipidemia (i.e., hypercholesterolemia, hypertriglyceridemia), and inflammation associated with CVD. In addition to CVD, risks associated with other diseases such as atherosclerosis, diabetes, cancer and other inflammatory conditions may potentially be reduced as well with the consumption of these vegetables. Results of this study contribute to the emergent body of evidence supporting the additive and synergistic contributions of dietary constituents such as dietary fibers, antioxidants, bioactive compounds and fatty acids, to health promotion and disease prevention. Future research studies may want to consider the inclusion of measurements of additional inflammatory cytokines (e.g., IL-6, IL-1, TNF-, etc.), endothelial function, genotypic and phenotypic modifications, lipid metabolism and aggregate cardiometabolic effects of collard greens, purslane and sweetpotato greens. 

## Figures and Tables

**Table 1:** Ingredient composition of experimental diets.

Ingredient (%)	Dietary Group *				
	AIN-76A	Control	CG	PL	SPG
Sucrose	50.00	41.96	39.27	39.49	39.39
Casein (Vitamin Free)	20.00	18.00	16.82	16.53	16.68
Corn Starch	15.00	15.00	15.00	15.00	15.00
Powdered Cellulose	5.00	5.00	5.00	5.00	5.00
AIN-76 Mineral Mix	3.50	3.50	3.50	3.50	3.50
AIN-76 Vitamin Mix	1.00	1.00	1.00	1.00	1.00
DL-Methionine	0.30	0.30	0.30	0.30	0.30
Choline Bitartrate	0.20	0.20	0.20	0.20	0.20
Ethoxyquin ^†^	0.00	0.00	0.00	0.00	0.00
Corn Oil	5.00	12.06	11.96	12.01	11.97
Soybean oil		2.91	2.88	2.89	2.89
Fish Oil					
Cholesterol		0.07	0.07	0.07	0.07
Collard Greens			4.00		
Purslane				4.00	
Sweetpotato Greens					4.00

* CG, collard greens; PL, purslane; SPG, sweetpotato greens. ^†^ Ethoxyquin content = 0.0010%.

**Table 2:** Nutrient composition * of experimental diets.

Nutrient	Dietary Group				
	AIN-76A	Control	CG	PL	SPG
Energy, kcal/100 g	370	436	441	436	438
Carbohydrates, %	66.10	60.30	60.30	61.70	61.90
Protein, %	17.20	15.70	16.30	15.20	15.30
Total dietary fiber, %	5.95	5.62	7.20	7.41	7.45
Moisture, %	10.00	6.86	5.56	5.55	5.49
Ash, %	2.57	2.53	2.93	3.25	2.96
Total Fat, g/100g	4.10	14.70	14.90	14.20	14.30
SFAs	0.66	2.19	2.25	2.25	2.75
MUFAs	1.17	4.03	3.91	3.61	3.20
PUFAs	1.97	7.47	7.40	6.81	7.39
TFAs	0.05	0.16	0.50	0.82	0.09
Linoleic acid, %	49.40	51.20	49.30	46.6	39.10
Arachidonic acid, %	<0.10	<0.10	<0.10	<0.10	0.31
α-Linolenic acid, %	1.08	2.27	2.55	2.76	5.81
Eicosapentaenoic acid, %	<0.10	<0.10	<0.10	<0.10	3.68
Docosahexaenoic acid, %	<0.10	<0.10	<0.10	0.21	1.69

* CG, collard greens; PL, purslane; SPG, sweetpotato greens; SFAs = saturated fatty acids, MUFAs = monounsaturated fatty acids, PUFAs = polyunsaturated fatty acids, TFAs = trans fatty acids.

**Table 3:** SHR dietary intake and body weight.

Dietary Group	Initial Dietary Intake (g)	Final Dietary Intake (g)	Total Dietary Intake (g)	Baseline Weight (g)	Initial Body Weight (g)	Final Body Weight (g)
AIN-76A	11.8 ± 0.4 ^a^	13.8 ± 1.6 ^a^	81.5 ± 3.3 ^a^	65.3 ± 5.0 ^a^	147.1 ± 8.0 ^a^	252.9 ± 9.1 ^a^
Control	11.7 ± 0.3 ^a^	13.6 ± 1.6 ^a^	80.4 ± 3.5 ^a^	66.4 ± 6.7 ^a^	146.5 ± 9.6 ^a^	284.7 ± 13.2 ^b^
CG	11.9 ± 0.4 ^a^	13.8 ± 1.6 ^a^	81.5 ± 3.4 ^a^	63.5 ± 8.0 ^a^	149.2 ± 11.9 ^a^	290.8 ± 9.9 ^b^
PL	11.6 ± 0.6 ^a^	14.1 ± 1.6 ^a^	80.6 ± 4.8 ^a^	60.9 ± 6.9 ^a^	143.5 ± 11.4 ^a^	285.6 ± 16.2 ^b^
SPG	11.8 ± 0.4 ^a^	13.9 ± 1.5 ^a^	81.0 ± 3.7 ^a^	65.8 ± 8.2 ^a^	148.7 ± 10.0 ^a^	285.3 ± 11.9 ^b^

Data are expressed as mean ± SEM of 10 SHRs per dietary group; Different superscript alphabet (i.e., a, b) within a column indicate statistical significance (*p* < 0.05) between dietary groups. Initial dietary intake- average dietary intake at week 1; Final dietary intake—average dietary intake at week 6; Total dietary intake—average total dietary intake, week 1–week 6; Initial body weight—average body weight at week 1; Final body weight—average body weight at week 6.

**Table 4:** Mean plasma adiponectin and hsCRP concentrations of SHRs consuming the AIN-76A diet and diets with a 25:1 ω-6/ω-3 FAR for 6 weeks.

Variable	Dietary Group				
	AIN-76A	Control	CG	PL	SPG
APN (μg/mL)	43.0 ± 1.7 ^a^	38.6 ± 1.6 ^ab^	35.1 ± 2.3 ^abc^	29.5 ± 2.8 ^c^	31.5 ± 4.0 ^bc^
hsCRP (μg/mL)	397.0 ± 52.5 ^a^	1092.2 ± 168.2 ^b^	1164.0 ± 209.2 ^b^	1452.0 ± 302.0 ^b^	1084.2 ± 87.9 ^b^

Data are expressed as mean ± SEM of 6 SHRs per dietary group; Different superscript alphabet (i.e., a, b, c) within a row indicate statistical significance (*p* < 0.05) between dietary groups. APN, adiponectin; hsCRP, high sensitivity C-reactive protein.

**Table 5:** Plasma lipid profile of SHRs consuming the AIN-76A diet and diets with a 25:1 ω-6/ω-3 FAR for 6 weeks.

Variable	Dietary Group				
	AIN-76A	Control	CG	PL	SPG
TAG (mg/dL)	150.1 ± 7.2 ^a^	97.0 ± 4.2 ^c^	92.2 ± 7.3 ^c^	113.0 ± 4.0 ^bc^	118.4 ± 12.6 ^b^
TC (mg/dL)	75.5 ± 11.8 ^a^	64.1 ± 2.8 ^a^	62.5 ± 3.1 ^a^	61.6 ± 2.5 ^a^	58.0 ± 2.3 ^a^
HDL-C (mg/dL)	39.2 ± 2.5 ^a^	33.7 ± 2.6 ^a^	38.7 ± 1.9 ^a^	41.3 ± 1.3 ^a^	19.4 ± 6.6 ^b^
LDL-C + VLDL-C (mg/dL)	10.5 ± 3.6 ^a^	15.1 ± 1.2 ^a^	12.6 ± 1.5 ^a^	10.5 ± 0.6 ^a^	11.2 ± 0.98 ^a^

Data are presented as mean ± SEM of 10 SHRs per dietary group. Rows with different alphabetical superscripts (i.e., a, b, c) indicate statistical significance at *p* < 0.05 according to Duncan’s post hoc test values. TAG: triglyceride (mg/dL); TC: total cholesterol (mg/dL); HDL-C: high-density lipoprotein cholesterol (mg/dL); LDL-C + VLDL-C: low-density lipoprotein cholesterol + very low-density lipoprotein cholesterol (mg/dL).

## References

[1] Benjamin E.J., Virani S.S., Callaway C.W., Chamberlain A.M., Chang A.R., Cheng S., Chiuve S.E., Cushman M., Delling F.N., Deo R. (2018). Heart disease and stroke statistics—2018 update: A report from the American Heart Association. Circulation.

[2] Medina-Remón A., Kirwan R., Lamuela-Raventós R.M., Estruch R. (2018). Dietary patterns and the risk of obesity, type 2 diabetes mellitus, cardiovascular diseases, asthma, and neurodegenerative diseases. Crit. Rev. Food Sci. Nutr..

[3] Monge A., Lajous M., Ortiz-Panozo E., Rodríguez B.L., Góngora J.J., López-Ridaura R.J.N.J. (2018). Western and Modern Mexican dietary patterns are directly associated with incident hypertension in Mexican women: A prospective follow-up study. Nutr. J..

[4] Hojhabrimanesh A., Akhlaghi M., Rahmani E., Amanat S., Atefi M., Najafi M., Hashemzadeh M., Salehi S., Faghih S. (2017). A Western dietary pattern is associated with higher blood pressure in Iranian adolescents. Eur. J. Nutr..

[5] Fanelli Kuczmarski M., Bodt B.A., Stave Shupe E., Zonderman A.B., Evans M.K. (2018). Dietary Patterns Associated with Lower 10-Year Atherosclerotic Cardiovascular Disease Risk among Urban African-American and White Adults Consuming Western Diets. Nutrients.

[6] Drake I., Sonestedt E., Ericson U., Wallström P., Orho-Melander M. (2018). A Western dietary pattern is prospectively associated with cardio-metabolic traits and incidence of the metabolic syndrome. Br. J. Nutr..

[7] Oikonomou E., Psaltopoulou T., Georgiopoulos G., Siasos G., Kokkou E., Antonopoulos A., Vogiatzi G., Tsalamandris S., Gennimata V., Papanikolaou A. (2018). Western Dietary Pattern Is Associated With Severe Coronary Artery Disease. Angiology.

[8] Simopoulos A.P.J.B. (2006). Pharmacotherapy. Evolutionary aspects of diet, the omega-6/omega-3 ratio and genetic variation: Nutritional implications for chronic diseases. Biomed. Pharmacother..

[9] Saini R.K., Keum Y.-S. (2018). Omega-3 and omega-6 polyunsaturated fatty acids: Dietary sources, metabolism, and significance—A review. Life Sci..

[10] Simopoulos A. (1999). Essential fatty acids in health and chronic disease. Am. J. Clin. Nutr..

[11] Simopoulos A.P. (1991). Omega-3 fatty acids in health and disease and in growth and development. Am. J. Clin. Nutr..

[12] Fedacko J., Vargova V., Singh R.B., Anjum B., Takahashi T., Tongnuka M., Dharwadkar S., Singh S., Singh V., Kulshresth S.K. (2012). Association of high w-6/w-3 fatty acid ratio diet with causes of death due to noncommunicable diseases among urban decedents in North India. Open Nutr. J..

[13] Salas-Salvadó J., Becerra-Tomás N., García-Gavilán J.F., Bulló M., Barrubés L. (2018). Mediterranean Diet and Cardiovascular Disease Prevention: What Do We Know?. Prog. Cardiovasc. Dis..

[14] Schulze M.B., Martínez-González M., Fung T.T., Lichtenstein A.H., Forouhi N.G. (2018). Food based dietary patterns and chronic disease prevention. BMJ.

[15] Tsioufis C. (2018). The Mediterranean and the DASH dietary patterns: Insights into their role in cardiovascular disease prevention. Hellenic. J. Cardiol..

[16] Maddock J., Ziauddeen N., Ambrosini G.L., Wong A., Hardy R., Ray S. (2018). Adherence to a Dietary Approaches to Stop Hypertension (DASH)-type diet over the life course and associated vascular function: A study based on the MRC 1946 British birth cohort. Br. J. Nutr..

[17] Johnston C.S., Taylor C.A., Hampl J.S. (2000). More Americans are eating “5 a day” but intakes of dark green and cruciferous vegetables remain low. J. Nutr..

[18] Lee-Kwan S.H., Moore L.V., Blanck H.M., Harris D.M., Galuska D. (2017). Disparities in State-Specific Adult Fruit and Vegetable Consumption—United States, 2015. MMWR.

[19] Adams M.R., Golden D.L., Chen H., Register T.C., Gugger E.T. (2006). A diet rich in green and yellow vegetables inhibits atherosclerosis in mice. Food Chem..

[20] Chen G.-C., Koh W.-P., Yuan J.-M., Qin L.-Q., van Dam R.M. (2018). Green leafy and cruciferous vegetable consumption and risk of type 2 diabetes: Results from the Singapore Chinese Health Study and meta-analysis. Br. J. Nutr..

[21] Mori N., Shimazu T., Charvat H., Mutoh M., Sawada N., Iwasaki M., Yamaji T., Inoue M., Goto A., Takachi R. (2018). Cruciferous vegetable intake and mortality in middle-aged adults: A prospective cohort study. Clin. Nutr..

[22] Pollock R.L. (2016). The effect of green leafy and cruciferous vegetable intake on the incidence of cardiovascular disease: A meta-analysis. JRSM Cardiovasc. Dis..

[23] Huang Z., Wang B., Eaves D., Shikany J., Pace R. (2007). Phenolic compound profile of selected vegetables frequently consumed by African Americans in the southeast United States. Food Chem..

[24] Huang Z., Wang B., Eaves D., Shikany J., Pace R.D. (2009). Total phenolics and antioxidant capacity of indigenous vegetables in the southeast United States: Alabama Collaboration for Cardiovascular Equality Project. Int. J. Food Sci. Nutr..

[25] Mohamed A.I., Hussein A.S. (1994). Chemical composition of purslane (*Portulaca oleracea*). Plant Foods Hum. Nutr..

[26] Petropoulos S.A., Karkanis A., Fernandes A., Barros L., Ferreira I.C., Ntatsi G., Petrotos K., Lykas C., Khah E. (2015). Chemical Composition and Yield of Six Genotypes of Common Purslane (*Portulaca oleracea* L.): An Alternative Source of Omega-3 Fatty Acids. Plant Foods Hum. Nutr..

[27] Almazan A.M., Begum F., Johnson C. (1997). Nutritional quality of sweetpotato greens from greenhouse plants. J. Food Compos. Anal..

[28] Almazan A.M., Adeyeye S.O. (1998). Fat and fatty acid concentrations in some green vegetables. J. Food Compos. Anal..

[29] Lin L.-Z., Harnly J.M. (2009). Identification of the Phenolic Components of Collard Greens, Kale, and Chinese Broccoli. Agric. Food. Chem..

[30] Johnson M., Pace R. (2010). Sweet potato leaves: Properties and synergistic interactions that promote health and prevent disease. Nutr. Rev..

[31] Oduro I., Ellis W.O., Owusu D. (2008). Nutritional potential of two leafy vegetables: Moringa oleifera and Ipomoea batatas leaves. Sci. Res. Essay.

[32] Joshipura K.J., Hu F.B., Manson J.E., Stampfer M.J., Rimm E.B., Speizer F.E., Colditz G., Ascherio A., Rosner B., Spiegelman D. (2001). The effect of fruit and vegetable intake on risk for coronary heart disease. Ann. Intern. Med..

[33] Blekkenhorst L.C., Bondonno C.P., Lewis J.R., Devine A., Zhu K., Lim W.H., Woodman R.J., Beilin L.J., Prince R.L., Hodgson J.M. (2017). Cruciferous and allium vegetable intakes are inversely associated with 15-year atherosclerotic vascular disease deaths in older adult women. J. Am. Heart Assoc..

[34] Blekkenhorst L.C., Bondonno C.P., Lewis J.R., Woodman R.J., Devine A., Bondonno N.P., Lim W.H., Zhu K., Beilin L.J., Thompson P.L. (2018). Cruciferous and total vegetable intakes are inversely associated with subclinical atherosclerosis in older adult women. J. Am. Heart Assoc..

[35] Duarte J., Pérez-Palencia R., Vargas F., Ocete M.A., Pérez-Vizcaino F., Zarzuelo A., Tamargo J. (2001). Antihypertensive effects of the flavonoid quercetin in spontaneously hypertensive rats. Br. J. Pharmacol..

[36] Edwards R.L., Lyon T., Litwin S.E., Rabovsky A., Symons J.D., Jalili T. (2007). Quercetin reduces blood pressure in hypertensive subjects. J. Nutr..

[37] Larson A.J., Symons J.D., Jalili T. (2012). Therapeutic Potential of Quercetin to Decrease Blood Pressure: Review of Efficacy and Mechanisms. Adv. Nutr..

[38] Serban M.C., Sahebkar A., Zanchetti A., Mikhailidis D.P., Howard G., Antal D., Andrica F., Ahmed A., Aronow W.S., Muntner P. (2016). Effects of quercetin on blood pressure: A systematic review and meta-analysis of randomized controlled trials. J. Am. Heart Assoc..

[39] Delgado C., Chertow G.M., Kaysen G.A., Dalrymple L.S., Kornak J., Grimes B., Johansen K.L. (2017). Associations of Body Mass Index and Body Fat With Markers of Inflammation and Nutrition Among Patients Receiving Hemodialysis. Am. J. Kidney Dis..

[40] Han S.J., Boyko E.J., Fujimoto W.Y., Kahn S.E., Leonetti D.L. (2017). Low Plasma Adiponectin Concentrations Predict Increases in Visceral Adiposity and Insulin Resistance. J. Clin. Endocrinol. Metab..

[41] Peri-Okonny P., Ayers C., Maalouf N., Das S.R., de Lemos J.A., Berry J.D., Turer A.T., Neeland I.J., Scherer P.E., Vongpatanasin W. (2017). Adiponectin protects against incident hypertension independent of body fat distribution: Observations from the Dallas Heart Study. Diabetes Metab. Res. Rev..

[42] Kyrou I., Tsantarlioti O., Panagiotakos D.B., Tsigos C., Georgousopoulou E., Chrysohoou C., Skoumas I., Tousoulis D., Stefanadis C., Pitsavos C. (2017). Adiponectin circulating levels and 10-year (2002–2012) cardiovascular disease incidence: The ATTICA Study. Endocrine.

[43] Furuhashi M., Ura N., Higashiura K., Murakami H., Tanaka M., Moniwa N., Yoshida D., Shimamoto K. (2003). Blockade of the renin-angiotensin system increases adiponectin concentrations in patients with essential hypertension. Hypertens.

[44] Cnop M., Havel P.J., Utzschneider K.M., Carr D.B., Sinha M.K., Boyko E.J., Retzlaff B.M., Knopp R.H., Brunzell J.D., Kahn S.E. (2003). Relationship of adiponectin to body fat distribution, insulin sensitivity and plasma lipoproteins: Evidence for independent roles of age and sex. Diabetologia.

[45] Dehghan F., Soori R., Gholami K., Abolmaesoomi M., Yusof A., Muniandy S., Heidarzadeh S., Farzanegi P., Ali azarbayjani M. (2016). Purslane (Portulaca oleracea) Seed Consumption And Aerobic Training Improves Biomarkers Associated with Atherosclerosis in Women with Type 2 Diabetes (T2D). Sci. Rep..

[46] Nazeam J.A., El-Hefnawy H.M., Omran G., Singab A.-N. (2018). Chemical profile and antihyperlipidemic effect of Portulaca oleracea L. seeds in streptozotocin-induced diabetic rats. Nat. Prod. Res..

[47] Soori R., Shahedi V., Akbarnejad A., Choobineh S. (2018). Biochemical changes in oxidative stress markers following endurance training and consumption of purslane seed in rats with hydrogen peroxide-induced toxicity. Sport Sci. Health.

[48] Hussein M.A. (2010). Purslane extract effects on obesity-induced diabetic rats fed a high-fat diet. Malaysian J. Nutr..

[49] Gray B., Steyn F., Davies P.S.W., Vitetta L. (2013). Omega-3 fatty acids: A review of the effects on adiponectin and leptin and potential implications for obesity management. Eur. J. Clin. Nutr..

[50] DeClercq V., d'Eon B., McLeod R.S. (2015). Fatty acids increase adiponectin secretion through both classical and exosome pathways. Biochim. Biophys. Acta Mol. Cell. Biol. Lipids.

[51] Simopoulos A. (2016). An Increase in the Omega-6/Omega-3 Fatty Acid Ratio Increases the Risk for Obesity. Nutrients.

[52] Hu E., Liang P., Spiegelman B.M. (1996). AdipoQ Is a Novel Adipose-specific Gene Dysregulated in Obesity. J. Biol. Chem..

[53] Asayama K., Hayashibe H., Dobashi K., Uchida N., Nakane T., Kodera K., Shirahata A., Taniyama M. (2003). Decrease in serum adiponectin level due to obesity and visceral fat accumulation in children. Obes. Res..

[54] Fontana L., Eagon J.C., Trujillo M.E., Scherer P.E., Klein S. (2007). Visceral fat adipokine secretion is associated with systemic inflammation in obese humans. Diabetes.

[55] Esposito K., Nappo F., Giugliano F., Di Palo C., Ciotola M., Barbieri M., Paolisso G., Giugliano D. (2003). Meal modulation of circulating interleukin 18 and adiponectin concentrations in healthy subjects and in patients with type 2 diabetes mellitus. Am. J. Clin. Nutr..

[56] Pischon T., Girman C.J., Rifai N., Hotamisligil G.S., Rimm E.B. (2005). Association between dietary factors and plasma adiponectin concentrations in men. Am. J. Clin. Nutr..

[57] Peake P.W., Kriketos A.D., Denyer G.S., Campbell L.V., Charlesworth J.A. (2003). The postprandial response of adiponectin to a high-fat meal in normal and insulin-resistant subjects. Int. J. Obes..

[58] Lithander F.E., Keogh G.F., Wang Y., Cooper G.J.S., Mulvey T.B., Chan Y.-K., McArdle B.H., Poppitt S.D. (2008). No evidence of an effect of alterations in dietary fatty acids on fasting adiponectin over 3 weeks. Obesity.

[59] Huang T., Tobias D.K., Hruby A., Rifai N., Tworoger S.S., Hu F.B. (2016). An Increase in Dietary Quality Is Associated with Favorable Plasma Biomarkers of the Brain-Adipose Axis in Apparently Healthy US Women. J. Nutr..

[60] Suzuki K., Inoue T., Hashimoto S., Ochiai J., Kusuhara Y., Ito Y., Hamajima N.J. (2010). Association of serum carotenoids with high molecular weight adiponectin and inflammation markers among Japanese subjects. Clin. Chim. Acta.

[61] Saura-Calixto F. (2010). Dietary fiber as a carrier of dietary antioxidants: An essential physiological function. J. Agric. Food Chem..

[62] Ntalla I., Dedoussis G., Yannakoulia M., Smart M.C., Louizou E., Sakka S.D., Papoutsakis C., Talmud P. (2009). ADIPOQ gene polymorphism rs1501299 interacts with fibre intake to affect adiponectin concentration in children: The GENe–Diet Attica Investigation on childhood obesity. Eur. J. Nutr..

[63] Hermsdorff H.H.M., Puchau B., Volp A.C.P., Barbosa K.B.F., Bressan J., Zulet M.Á., Martínez J.A. (2011). Dietary total antioxidant capacity is inversely related to central adiposity as well as to metabolic and oxidative stress markers in healthy young adults. Nutr. Metab..

[64] Devaraj S., Torok N., Dasu M.R., Samols D., Jialal I. (2008). Adiponectin decreases C-reactive protein synthesis and secretion from endothelial cells: Evidence for an adipose tissue-vascular loop. Arterioscler. Thromb. Vasc. Biol..

[65] Detopoulou P., Panagiotakos D., Chrysohoou C., Fragopoulou E., Nomikos T., Antonopoulou S., Pitsavos C., Stefanadis C. (2010). Dietary antioxidant capacity and concentration of adiponectin in apparently healthy adults: The ATTICA study. Eur. J. Clin. Nutr..

[66] Franzini L., Ardigo D., Valtuena S., Pellegrini N., Del Rio D., Bianchi M., Scazzina F., Piatti P., Brighenti F., Zavaroni I. (2012). Food selection based on high total antioxidant capacity improves endothelial function in a low cardiovascular risk population. Nutr. Metab. Cardiovasc. Dis..

[67] Kamigaki M., Sakaue S., Tsujino I., Ohira H., Ikeda D., Itoh N., Ishimaru S., Ohtsuka Y., Nishimura M. (2006). Oxidative stress provokes atherogenic changes in adipokine gene expression in 3T3-L1 adipocytes. Biochem. Biophys. Res. Commun..

[68] D’Archivio M., Filesi C., Di Benedetto R., Gargiulo R., Giovannini C., Masella R. (2007). Polyphenols, dietary sources and bioavailability. Ann. Ist. Super Sanità.

[69] D’Archivio M., Filesi C., Varì R., Scazzocchio B., Masella R. (2010). Bioavailability of the polyphenols: Status and controversies. Int. J. Mol. Sci..

[70] Kahlon T., Chapman M., Smith G. (2007). In vitro binding of bile acids by spinach, kale, brussels sprouts, broccoli, mustard greens, green bell pepper, cabbage and collards. Food Chem..

[71] Kahlon T.S., Chiu M.-C.M., Chapman M.H. (2008). Steam cooking significantly improves in vitro bile acid binding of collard greens, kale, mustard greens, broccoli, green bell pepper, and cabbage. Nutr. Res..

[72] Innami S., Tabata K., Shimizu J., Kusunoki K., Ishida H., Matsuguma M., Wada M., Sugiyama N., Kondo M. (1998). Dried green leaf powders of Jew‘s mellow (Corchorus), persimmon (Diosphyros kaki) and sweet potato (Ipomoea batatas poir) lower hepatic cholesterol concentration and increase fecal bile acid excretion in rats fed a cholesterol-free diet. Plant Foods Hum. Nutr..

[73] Sadakane A., Tsutsumi A., Gotoh T., Ishikawa S., Ojima T., Kario K., Nakamura Y., Kayaba K. (2008). Dietary patterns and levels of blood pressure and serum lipidsin a japanese population. J. Epiddemiol..

[74] Gallaher D.D., Hassel C.A., Lee K.-J., Gallaher C.M. (1993). Viscosity and fermentability as attributes of dietary fiber responsible for the hypocholesterolemic effect in hamsters. J. Nutr..

[75] Brown L., Rosner B., Willett W.W., Sacks F.M. (1999). Cholesterol-lowering effects of dietary fiber: A meta-analysis. Am. J. Clin. Nutr..

[76] Estruch R., Martínez-González M.A., Corella Piquer D., Basora-Gallisá J., Ruiz-Gutiérrez V., Covas Planells M.I., Fiol Sala M., Gómez Gracia E., López Sabater M.C. (2009). Effects of dietary fibre intake on risk factors for cardiovascular disease in subjects at high risk. J. Epidemiol. Community Health.

[77] Anderson J.W., Baird P., Davis R.H., Ferreri S., Knudtson M., Koraym A., Waters V., Williams C.L. (2009). Health benefits of dietary fiber. Nutr. Rev..

[78] Jacobs D.R., Mebane I.L., Bangdiwala S.I., Criqui M.H., Tyroler H.A. (1990). High density lipoprotein cholesterol as a predictor of cardiovascular disease mortality in men and women: The follow-up study of the Lipid Research Clinics Prevalence Study. Am. J. Epidemiol..

[79] Johnson M., Pace R.D., McElhenney W. (2018). Green leafy vegetables in diets with a 25:1 omega-6/omega-3 fatty acid ratio modify the erythrocyte fatty acid profile of spontaneously hypertensive rats. Lipids Health Dis..

[80] Balk E., Lichtenstein A.H., Chung M., Kupelnick B., Chew P., Lau J. (2006). Effects of omega-3 fatty acids on serum markers of cardiovascular disease risk: A systematic review. Atherosclerosis.

[81] Harris W.S., Miller M., Tighe A.P., Davidson M.H., Schaefer E.J. (2008). Omega-3 fatty acids and coronary heart disease risk: Clinical and mechanistic perspectives. Atherosclerosis.

[82] Lundberg J.O., Carlström M., Weitzberg E. (2018). Metabolic effects of dietary nitrate in health and disease. Cell Metab..

[83] van Breda S.G.J., de Kok T.M.C.M. (2018). Smart Combinations of Bioactive Compounds in Fruits and Vegetables May Guide New Strategies for Personalized Prevention of Chronic Diseases. Mol. Nutr. Food Res..

[84] Hadi A., Pourmasoumi M., Najafgholizadeh A., Kafeshani M., Sahebkar A. (2019). Effect of purslane on blood lipids and glucose: A systematic review and meta-analysis of randomized controlled trials. Phytother. Res..

[85] Sun H., Mu B., Song Z., Ma Z., Mu T. (2018). The In Vitro Antioxidant Activity and Inhibition of Intracellular Reactive Oxygen Species of Sweet Potato Leaf Polyphenols. Oxid. Med. Cell Longev..

[86] Mozaffarian D., Wu J.H.Y. (2011). Omega-3 Fatty Acids and Cardiovascular Disease: Effects on Risk Factors, Molecular Pathways, and Clinical Events. J. Am. Coll. Cardiol..

[87] Trayhurn P., Wood I.S. (2004). Adipokines: Inflammation and the pleiotropic role of white adipose tissue. Br. J. Nutr..

